# Medical History from the Earliest Times

**Published:** 1893-01-21

**Authors:** E. T. Withington


### MEDICAL HISTORY FROM THE EARLIEST TIMES.

By E. T. WITHINGTON, M.A., M.B. Oxon.

### XXXIX.—THE ARABO-SCHOLASTIC REVIVAL. (2) SURGERY.

GUY OF CHAULIAC, "Restorer of Surgery," gives us in the preface to his chief work a brief estimate of his immediate predecessors in that art. Till the time of Avicenna physic and surgery were, he says, united, but since then they became separate, and the latter degenerated into the hands of mechanics. "The earliest of these were Roger, Roland, and the 'Four Masters,' who wrote special books on surgery, in which they mixed up much empiricism. Later came Bruno, who conbined cleverly enough the theories of Galen and Avicenna with the practice of Albucasis, but he had no complete translation of Galen, and he has entirely omitted anatomy. Then came Theodoric, who compiled a book by stealing everything Bruno had said, with some fables of his master, Hugh of Lucca. William of Saliceto was a man of ability (*valens homo*); he composed two epitomes of physic and surgery, and in my opinion treated those subjects very tolerably, so far as he went. Lanfranc also wrote a book containing little else than what he got from William, but he changed the order. At the same time, Magister Arnaldus of Villanova flourished in both faculties, and wrote many excellent works." He afterwards divides them into three sects, according to their mode of treating wounds. (1) Roger, Roland, and the Four Masters treated all wounds and inflammations with poultices; (2) Bruno and Theodoric used wine only and dry dressings; while (3) William of Saliceto and Lanfranc, wishing to mediate between the two, cured all wounds with ointments and sedative plasters. Guy's criticisms are, perhaps, sometimes too severe, especially in the case of Lanfranc, and his divisions are rather too arbitrary, for all the above-mentioned surgeons used ointments, though those of the Salernitan school direct them to be put round not on the wound, "for Constantine declares that fatty substances are not good for wounds."

The fathers of Italian and indirectly of European surgery were Roger of Parma and Hugh of Lucca, of whom the former is known as a writer and the latter as a practitioner only. Roger studied and taught at Salerno, and his work, the "Rogerina" (1186), which was edited by his pupil, Roland, and commented upon by the mysterious Four Masters, long formed the Salernitan text-book of surgery. Of special interest is his recommendation of the ashes of sponge and seaweed, which would, of course, contain iodides, for goitre, and the commentary of the Four Masters contains one of the earliest examples of a "post mortem." Speaking of the difficulties in treating head injuries, they relate: "A young man was struck on the head with a stone from a sling; he presented no symptoms, but the next day he was found dead, and, on opening the skull, a large blood clot was seen on the surface of the dura mater."

Hugh of Lucca belonged to the noble family of Bourgognone, one of whom was prætor or podesta of Bologna in 1214, when Hugh was appointed city surgeon. The agreement still exists in which he binds himself, for a single payment of 600 lira, to reside during half the year in the city, to attend the poor gratis, and to accompany military expeditions, besides which he was afterwards required to give evidence in medico-legal matters. His name is honourably connected with the early history of anæsthetics, which may be briefly sketched here. It is often asserted, and it is not impossible, that the ancient surgeons sometimes produced insensibility by means of hypnotism; but there is no direct evidence on the subject. They appear, however, to have sometimes obtained a local and partial anæsthesia by the application of a "lapis memphiticus," perhaps a kind of bitumen, which acted through the phenol it contained, and they certainly used decoctions of poppy and mandragora, as did also the mediæval operators. Bernard tells us that the Salernitans rubbed up poppy seed and henbane, and used them as a plaster to deaden the sensibility of a part to be cauterised. The author of the "Breviarium" (Arnald of Villanova ?) gives the following recipe: "To produce sleep so profound that the patient may be cut and will feel nothing, as though he were dead. It is an "experimentum" of Magister Michael Scot. Take of opium, mandragora bark, and henbane root equal parts, pound them together, and mix with water. When you want to sew or cut a man dip a rag in this and put it to his forehead and nostrils. He will soon sleep so deeply that you may do what you will. To wake him up, dip the rag in strong vinegar. The same is excellent in brain fever, when the patient cannot sleep, for if he do not sleep he will die." Hugh of Lucca's method was either the original or an improved version of this. He added the juice of lettuce, ivy, mulberry, sorrel, and hemlock to the above, and boiled the whole with a new sponge. This was then dried, and when wanted, dipped in hot water and applied to the patient's nostrils.

What we know of the practice of Hugh of Lucca is gathered from the writings of his son Theodoric, Bishop of Cervia, and surgeon and "poenitentiarius" to Pope Innocent IV. He is remarkably fond of mercurial ointments, especially an "unguentum saracenicum." Besides parasitic skin affections this is, he says, the best remedy for the "malum mortuum," a disease characterised by chronic ulcers on the arms and legs, and for some forms of rheumatism, while it will even cure leprosy in the early stage. The patient should stand between two fires and rub his arms and legs daily with the ointment till he produces toothache and salivation.

But the greatest surgeons of the second half of the thirteenth century were William of Saliceto and his pupil Lanfranc of Milan, both of whom frequently give their own experience, as well as that of their predecessors. We may accept Guy's estimate of the former, and space only permits two brief extracts from his surgical and medical work respectively: "In a certain army in which I served—and I was very young then—I saw a soldier of Bergamo struck by a great arrow from a machine. The arrow went in on the right side of his neck near the vessels, but without injuring them, and came out over his left shoulder. I extracted it in the way described in the chapter 'On Arrow Wounds of the Head,' and treated the wound in the manner often related. The man got perfectly well, and lived long after, and I got a good fee." "Hardness of the kidneys (durities renum). This may either be the result of an inflammation, or may come of itself, . . . and it is worse than other diseases, for it is difficult or impossible to cure."

It was Lanfranc who introduced the Italian surgery into France, and Guy's decidedly unfair estimate of him is, perhaps, a return for his criticisms of the French surgeons, who, he declares, were mostly "idiotæ," and utterly ignorant, not even knowing the distinction of the actual and potential cautery. He was, however, delighted with Paris, the earthly paradise, the city *sine pari*, and he was received with honour by the medical faculty, and by the Surgical College of St. Côme, of which he became a member A.D. 1295. It was partly to show his gratitude for this welcome that he wrote his "Ars Completa Totius Chirurgiæ." Among the many things which attract our notice in this work, his remarks on the treatment of hæmorrhage are perhaps the most interesting. Pressure is the first and best method; but he is acquainted with ligature and even torsion. "A boy at Milan, fifteen years old, was stabbed in the arm by another boy. The bleeding could not be stopped by the ordinary methods, so I judged it necessary to dissect out the vessel and tie it. The boy's mother, however, sent for a certain 'laicus,' who utterly reprobated my opinion, and promised to cure the patient. He stayed, and I departed; but the bleeding continued off and on till the patient was nearly dead. I was sent for, but declined to go. Then a physician acquainted with the patient's friends rebuked them and the boy's mother for rejecting my advice for that of the 'idiota,' and told them it was the only way to save his life. A surgeon was asked if he could perform what I had recommended, and replied he could, so he incised the skin over the vessel, 'et illam contorsit in manibus, et ligavit cum filo.'"

